# Exploring Surface-Enhanced Raman Spectroscopy of Pyrazine-2-Carbonitrile for Indirect Label-Free Albumin Quantification in an *In Vitro* Endothelium Permeability Assay

**DOI:** 10.1021/acs.analchem.4c05906

**Published:** 2025-02-10

**Authors:** W. J. Niels Klement, Daniël R. Duijnstee, Vika Telle, Aleksandar Staykov, Wesley R. Browne, Elisabeth Verpoorte

**Affiliations:** † Molecular Inorganic Chemistry, Stratingh Institute for Chemistry, Faculty of Science and Engineering, University of Groningen, Nijenborgh 3, 9474AG Groningen, The Netherlands; ‡ Pharmaceutical Analysis, Groningen Research Institute of Pharmacy, University of Groningen, Antonius Deusinglaan 1, 9713AV Groningen, The Netherlands; § International Institute for Carbon Neutral Energy Research (WPI-I_2_CNER), Kyushu University, Fukuoka 819-0395, Japan

## Abstract

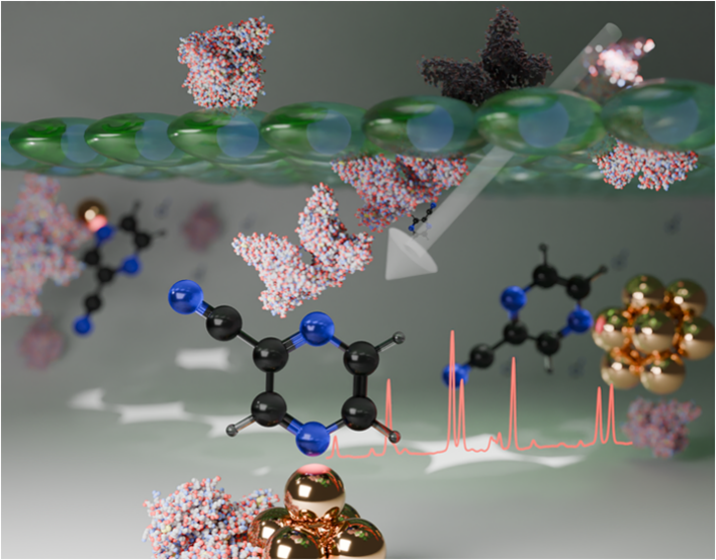

Accurate, label-free quantification of proteins, and more specifically albumin, is essential in studies aimed at monitoring transport across biological barrier tissues *in vitro*. Surface-enhanced Raman scattering (SERS) can deliver the sensitivity and specificity needed for such studies at physiologically relevant conditions, however, direct detection of albumin is not typically feasible at such concentrations. Here we use a small-molecule reporter (pyrazine-2-carbonitrile, PCN) that can interact both with albumin and a SERS substrate to facilitate albumin quantification. The nanoparticle surface/PCN and albumin/PCN interactions are sufficiently balanced to yield the sensitivity and specificity needed for *in vitro* tissue studies. The major challenge in using SERS for such assays is that the spectra of analytes can differ from their nonresonant Raman spectra, due to distinct species forming at and near the surface of the nanoparticles. Specifically, the binding of PCN to gold nanoparticles, formation of Au-PCN complexes, as well as PCN itself, contribute to the SERS spectra. We elucidate the nature of these interactions through concentration dependence studies and computational methods. Ultimately, we show that understanding these different interactions is key to quantification of albumin, at physiologically relevant albumin concentrations ranging from 0.4 to 4.4 μM using SERS spectroscopy. These data compare well with the state-of-the-art spectroscopic method, i.e., the transport of fluorescently labeled albumin across cell layers. We anticipate that this assay will stimulate analysis in in vitro models, such as organ-on-a-chip models and flow systems.

## Introduction

The endothelium is a layer of cells lining all blood and lymphatic vessels, collectively the vascular system, in the body. It forms a dynamic barrier with tight intercellular junctions that can open to allow for the selective passage of water and other small ionic and molecular species (up to a radius of 3.6 nm) from the vessel to the interstitial space between cells in surrounding tissue. Larger proteins cannot pass through intercellular junctions, but can be transported through endothelial cells by means of encapsulation in vesicles.^1^ A crucial protein in human physiology is albumin (66.5 kDa), which makes up about half of blood plasma protein.^2^ This protein is made by the liver, with most of the daily 10–15 g formed being delivered directly into the blood. Once there, albumin binds both endogenous molecular species as well as drugs and other exogenous species, and subsequently carries these species through the circulatory system. Of the albumin produced daily, 30–40% will remain in the vascular system, with the rest permeating the endothelium and vascular walls to end up in the interstitial space. Patients with diabetes, obesity, or chronic kidney disease experience enhanced albumin excretion, or albuminuria, from blood vessels, which have become more permeable due to disease. Modeling permeation of the endothelial barrier by proteins in vivo is therefore of interest in assessing endothelial dysfunction in these and other diseases.^3^

Over the past decade, microfluidic devices that can recreate the cellular barriers that separate compartments in the body, such as the endothelium, have seen widespread use in mimicking physiological functions and assessing barrier integrity.^4^ So called endothelium-on-a-chip models,^5,6^ for example, are constructed using a cultured endothelial cell layer on a porous membrane to separate an apical chamber (representing the blood vessel lumen) from a basolateral chamber (representing the tissue in contact with the outside of the blood vessel). The integrity of the barrier in regard to the passage of molecules, and in particular proteins, can be determined through changes in the electrical impedance of endothelial layers, or by macromolecular tracer flux assay.^7,8^ In the latter case, the amount of a, generally fluorescently labeled, macromolecular species that permeates the endothelial cell layer is quantified.^5,6^

As mentioned above, albumin’s passage through endothelium layers is a key marker for layer health. In vitro, albumin labeled with fluorophores is frequently used to track endothelium permeation, as it allows for optical detection.^9^ Fluorescence tracking is effective, but requires pretreatment of the albumin with labels, which at the very least adds a step to the in vitro analysis and in some cases is not feasible at all. For example, levels of albumin produced directly by liver tissue are a measure for hepatic function (e.g., to determine the hepatoxicity of drugs, etc.) but cannot be monitored using fluorescence spectroscopy, as the liver does not produce labeled albumin.^10^ A label-free method for quantitative albumin detection would thus open further possibilities especially in organ-on-a-chip applications, where in vitro label-free monitoring of albumin permeation in real time is desirable.

Nonspectroscopic (semi)quantitative methods for albumin detection for diagnostic purposes (i.e., albuminuria) include dip sticks, immunoassays,^11^ and gel electrophoresis. Dip sticks are read out visually through color changes. More quantitative methods include HPLC, mass spectrometry, or, when high throughput is desired, immunoassays such as ELISA. Immunoassays were used by Asif et al., in combination with electrochemical detection, to determine albumin production in a liver-on-a-chip.^10^

Raman spectroscopy is a label-free technique for quantitative spectroscopic detection of analytes, which does not require, for example, addition of fluorescent moieties to the analyte of interest.^12^ However, the concentration range over which compounds can be detected with Raman spectroscopy is limited (at least >1–10 mM), which constrains its practical use in analysis. Indeed, selective signal enhancement mechanisms are necessary for Raman spectroscopy to be used directly at the low analyte concentrations (μM to nM) typical of physiologically relevant liquid samples. Surface-enhanced Raman scattering (SERS) is of particular interest and is achieved through contact of analytes with the surface plasmon of a metallic substrate, such as between aggregated colloidal gold or silver nanoparticles, or an atomically rough noble metal surface, usually silver or gold.^13^ SERS spectroscopy can provide high sensitivity,^13,14^ in some cases even at the single-molecule level.^15^ It has found application in fields ranging from electrochemistry^16^ to biosensing,^17,18^ such as protein analysis,^19^ including albumin.^20,21^ SERS analysis of proteins, however, is generally challenging due to the absence of moieties with large Raman scattering cross sections.^20,22^ In biological samples, the presence of small biomolecules, such as adenine, coupled with the high sensitivity of SERS to these compounds, can result in spectra cluttered by Raman scattering from species other than those of interest.^23,24^ Furthermore, the relative enhancement factors of structurally similar compounds can differ significantly, which can potentially limit repeatability,^25^ as discussed recently in detail by Langer et al.^13^

Proteins, and especially albumin, consist mainly of aliphatic, nonaromatic, amino acids that have low Raman scattering cross sections,^22^ and hence require relatively high concentrations for analysis by SERS,^20,26^ as well as nonresonant Raman spectroscopy.^27^ SERS shows a strong dependence on distance from the surface, i.e., analytes must be close (within 1–2 nm) for significant enhancement.^13,28^ Detection with SERS at lower, physiologically relevant, concentrations can be achieved using surface-immobilized antibodies to hold albumin close to a SERS substrate.^19,29^

**Figure 1:**
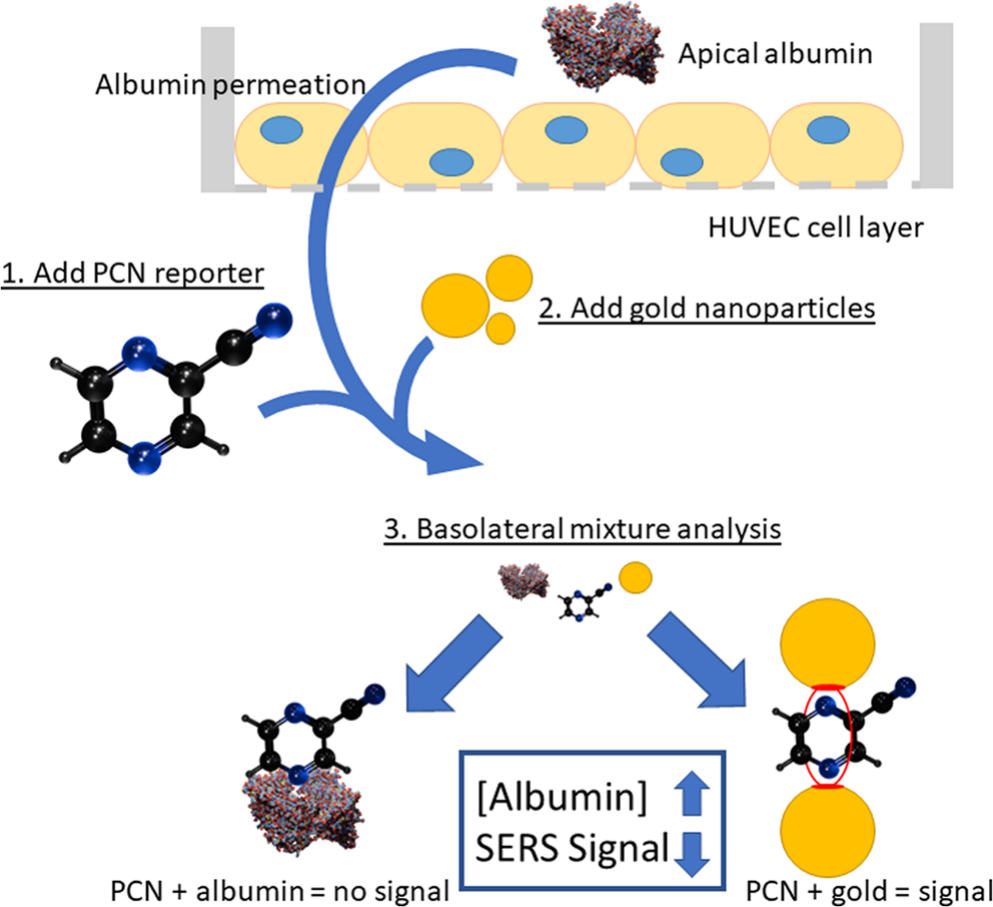
Albumin permeates through an endothelial cell layer cultured on a porous polymer Transwell membrane to enter the solution on the basolateral side. This solution is collected after a particular time, and PCN is added, which interacts/binds to the albumin in solution. Subsequently, gold nanoparticles are added. PCN interacts competitively with albumin and aggregating gold nanoparticles. Interaction with gold results in a SERS enhancement of the Raman scattering of PCN. The interaction of PCN with albumin suppresses that signal.

Albumin’s functions include maintaining osmotic pressure in the body, and facilitating transport of small molecules, hormones and pharmaceuticals around the circulatory system. In this study, we take advantage of this latter role in an indirect approach for the detection of albumin. Rather than detect albumin directly with SERS, we make use of a small-molecule reporting compound, pyrazine-2-carbonitrile (PCN), that can bind to albumin, as well as interact strongly with gold surfaces that provide large SERS enhancements, Figure 1. The dual role of the reporter is to produce a clear Raman signal upon interaction with a SERS-active substrate (gold nanoparticles between 10–50 nm), as well as to bind with albumin. Binding to albumin suppresses the Raman scattering of the reporter by holding it away from the SERS substrate (Figure 1). Thus, the more albumin in solution, the lower the SERS intensity from PCN.

However, the interaction of PCN with gold nanoparticle surfaces is not trivial. We show, using multivariate curve resolution (MCR) and density functional theory (DFT), that both PCN on its own and two distinct PCN-based species formed by chemical interaction with the gold substrate, contribute to the SERS spectra obtained. Understanding the relative contributions of each of these species to the SERS spectra is essential for preparation of a calibration curve for albumin quantification.

Finally, the competitive approach using a SERS reporter is applied to quantify albumin permeation of an endothelial cell barrier cultivated on the porous membrane of a transwell insert in a well plate, Figure 1, as a proof-of-concept necessary toward future application in an endothelium-on-a-chip devices.

## Experimental Section

### Materials

Bovine serum albumin (BSA), albumin-FITC (labeled Bovine Serum Albumin–fluorescein isothiocyanate conjugate), pyrazine-2-carbonitrile (PCN) and other chemicals were obtained from Sigma-Aldrich and used as received. A detailed description of the albumin solutions used in cell studies, as well as for reference and control experiments, can be found in the SI, page S3. Gold nanoparticles were prepared by adaptation of the citrate reduction method first reported by G. Frens in 1973 as described in the SI, Pages S3–S4.^30,31^

### Instrumentation

Raman spectra were recorded using a Raman probe (Avantes, The Netherlands), which was fiber-coupled with a 785 nm laser (500 mW, Cobolt, Hubner Photonics, Sweden) and a Shamrock163i spectrograph equipped with an iVac-316 LDC-DD CCD camera (Andor Oxford Instruments, UK). Spectra were calibrated with cyclohexane, following ASTM standards (E2911-23). UV/vis absorbance spectra were recorded with a Specord210plus spectrophotometer (AnalytikJena). Fluorescence spectra were recorded in a 1 cm path length quartz fluorescence cuvette using a FS5 spectrofluorimeter (Edinburgh Instruments). Transmission electron microscopy (TEM) micrographs were obtained using a Phillips CM120 electron microscope with 120 kV on a copper/carbon sample plate.

### SERS Spectroscopy and Calibration Curve Preparation

Calibration solutions (for both Raman spectroscopy and fluorescence analysis) were prepared using a stock solution of (labeled or nonlabeled) BSA in PBS (phosphate-buffered saline) buffer. Dilutions were prepared by adding the required amounts of this stock to PBS to obtain a total volume of 950 μL (1000 μL for labeled albumin). Pyrazine-2-carbonitrile in PBS (50 μL) was added to the unlabeled albumin solutions to obtain a total volume of 1000 μL, containing 50 μM pyrazine-2-carbonitrile and the desired BSA concentration. The solutions were mixed and equilibrated over 20 min. One mL of gold nanoparticle solution was then added, which changed the color of the solutions to a shade of red, Figure S2. A Raman spectrum was recorded immediately after mixing, and a second spectrum recorded after 15 min, to determine whether or not there were changes to the spectra over time. During mixing, the solution attained a slight blue/gray color, Figure S2, indicative of aggregation, which is a requirement for SERS enhancement using this type of SERS substrate.^25^ Despite the addition of the slightly acidic nanoparticle solution, the pH of the buffered solution was not changed.

### Density Functional Theory (DFT)

DFT calculations were performed using Turbomole 7.7 software with Perdew, Burke, Eindhoven (PBE) functional and def2-SV(P) basis set. The geometries of PCN adsorbed on gold nanoparticles with 1, 13, 55, and 147 atoms, and two possible symmetries: icosahedron and cuboctahedra, were optimized. Particles with cuboctahedra symmetry and sizes of 13, 55, and 147 atoms are denoted cub13, cub55, and cub147. Similarly, particles with icosahedron symmetry are denoted ico13, ico55, and ico147. The 147-atom nanoparticles have diameters of 1.8 nm, which is close in terms of properties to the scales of clusters expected to be observed experimentally.^32^ The geometries of the particle/PCN complexes were fully relaxed and frequency calculations were performed to confirm the ground state structures and to obtain frequencies of vibrational modes. Mulliken population analysis was performed to evaluate atomic charges and electron transfer between the nanoparticles and PCN. For each particle size, both symmetries were optimized. The symmetry with the lowest energy was used for further analyses.

### Cell Studies

**Figure 2:**
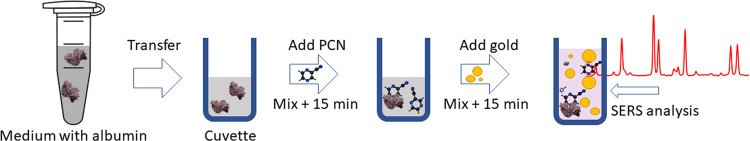
Sequence of analysis for albumin collected in the basolateral solution of HUVEC well plate culture. The basolateral medium is transferred to a 1 cm path length cuvette. PCN containing solution is added with mixing (50 μM final concentration). Some of the PCN will interact with albumin. After 15 min, gold nanoparticles are added. Gold interacts with the PCN that is not bound to albumin to provide SERS in the spectra recorded.

Human umbilical vein endothelial cells (HUVEC) were cultured to form confluent layers on Transwell inserts in well plates, as described in the SI, Pages S5–S7. During permeability assays, a solution of 0.2 mg/mL (44 μM) BSA in Hank’s balanced salt solution (HBSS) was added to the apical chamber (top side of the cell layer), which is approximately the concentration of albumin in the umbilical cord vein.^33,34^ The endothelial cell layer was incubated with this solution for 30 min to allow for endothelial layer permeation, after which the basolateral (lower compartment) liquid was collected for analysis. SERS analysis of BSA was carried out according to the protocol shown in Figure 2.

## Results and Discussion

The concentrations of albumin expected to permeate the endothelial layer cultured on the Transwell membrane were determined by an established fluorescence assay (Figure 1 and SI for details).^35^ In the arrangement used here, the transferred albumin was found to be ca. 8% (3.5 μM). The HUVEC culture used in the present study was chosen to facilitate comparison of the SERS-based method described here and the fluorescence-based assay described earlier. The data obtained here with the fluorescence-based method provided the concentration range that should be covered by a SERS method.

**Figure 3:**
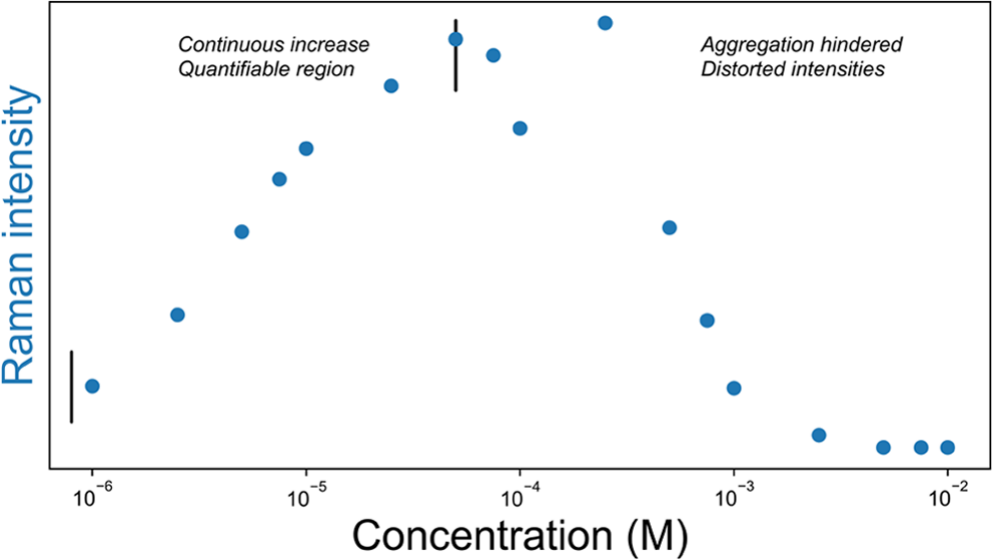
Dependence of SERS intensity on concentration of PCN, with constant volume of gold nanoparticle solution. Intensity was calculated from the integrated area of the band of PCN at 1200 cm^–1^. Intensity increases with increase in concentration until a critical point, where aggregation becomes hindered and ultimately the solution becomes opaque due to low solubility. Concentration range used in the analysis is indicated by vertical black lines.

Gold nanoparticles were used as SERS substrate, as the surface plasmon when aggregated shifts to the near-infrared, Figure S2, and hence, exhibits good overlap with excitation at 785 nm. Excitation at 785 nm was chosen to minimize interference from the background (auto)fluorescence typically observed from biological media. Gold nanoparticles can be aggregated by salts, such as those typically present in buffers or cell medium, turning on the SERS signal.^13,14,25,28^ The enhancement provided by the nanoparticles with PCN is observed over a relatively wide concentration range of up to 4 orders of magnitude, Figure 3.

### Determination of Relevant Concentration Range for PCN

The dependence of intensity on concentration is typically nonlinear for SERS due to several factors influencing intensity, such as the Langmuir isotherm (surface adsorption), aggregation, and enhancement factors that differ over the surface of the SERS substrate. Typically, the intensity/concentration dependence with aggregated nanoparticle-based SERS is manifested in a bell-shaped curve.

The Raman scattering of PCN shows this behavior, Figure 3. At low concentrations, the Raman intensity of PCN is logarithmically proportional to concentration. However, at higher concentrations (ca. 100 μM) the intensity ceases to increase, suggesting saturation of SERS hot spots, after which the Raman intensities decrease again. This decrease is attributed to the surface of the nanoparticles being coated in PCN, preventing aggregation, and is also consistent with a lack of color change in these samples (vide supra).

For the purposes of quantification, a relation between concentration and intensity is required and therefore, the concentration range of PCN useful for quantitative studies is below 100 μM. As there are also other species present in cell culture media that can interact with gold surfaces (vide infra),^36^ the concentration of PCN (50 μM) selected as a start point for the assay is well below this threshold. At this concentration, colloid aggregation is also not expected to be affected by PCN. More importantly, this concentration is the most sensitive to changes in physiologically relevant concentrations of albumin.

### Comparison of SERS and Raman Spectra of PCN

**Figure 4:**
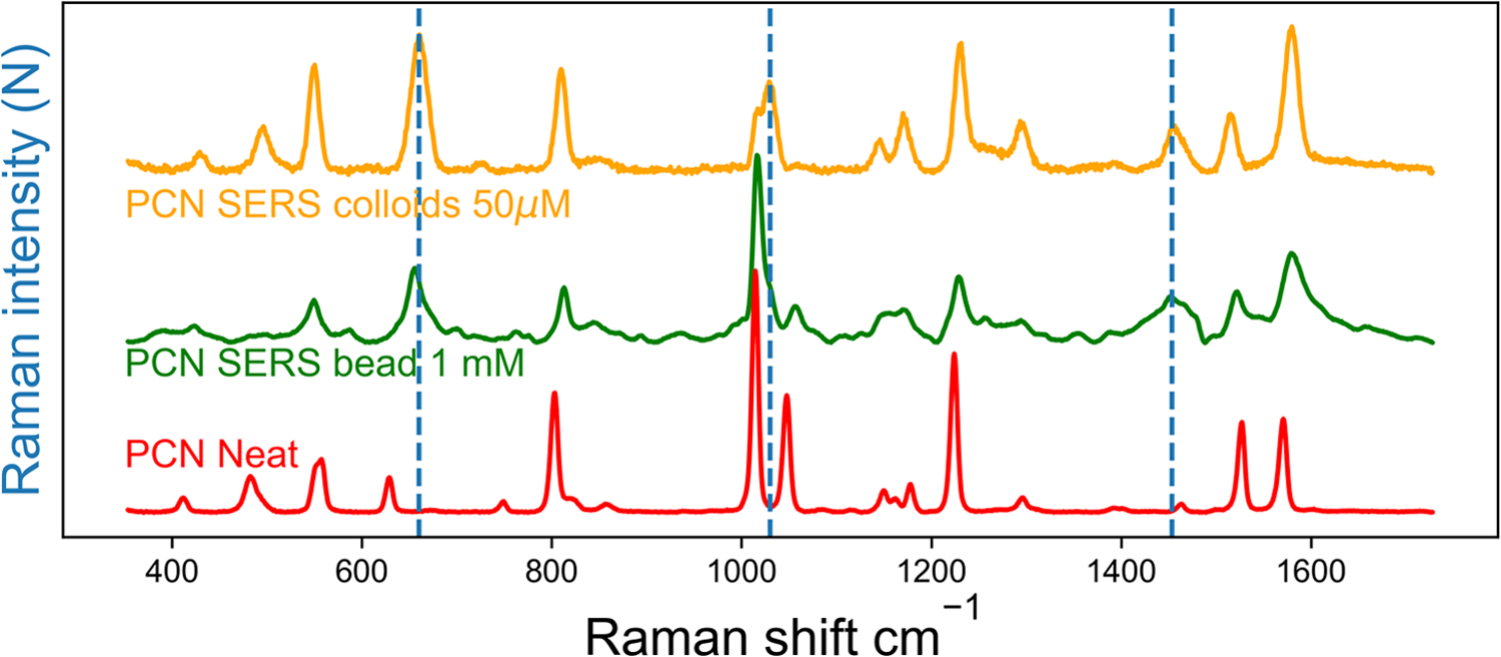
SERS spectra of PCN (yellow 50 μM) obtained with gold nanoparticles aggregated with PBS (5 mM), (green) using a roughened gold surface and (red) nonresonant Raman spectrum of neat PCN (excitation λ_exc_ 785 nm). Dashed blue lines indicate bands that show the most prominent shifts between nonresonant Raman and SERS spectra.

The Raman spectrum of neat (or aqueous) PCN shows substantial differences compared to its SERS spectrum, Figure 4. Since the Raman spectrum is a manifestation of molecular structure and shape, differences in number of bands and their position indicate a structural change. The nonresonant Raman spectrum of PCN is also dependent on its environment, as seen in the minor shifts in the bands when in aqueous solution, Figure S7. However, the additional bands and large shifts in band position observed in the SERS spectrum suggest a large structural change between neat or aqueous PCN, and PCN observed with surface enhancement. The nitrile stretching band of PCN appears at 2252 cm^–1^ in the neat liquid and is intense relative to the bands in the fingerprint region. In aqueous solution the band is shifted to lower wavenumber (2236 cm^–1^) and is slightly weaker relative to the fingerprint region. In contrast, the nitrile stretching band in the SERS spectrum of PCN is slightly broadened and much less intense compared to the bands in the fingerprint region. The decrease in relative intensity and the variability in peak positions mean that it is less useful for quantitative studies, despite the fact that the relative isolation of the band is in some aspects attractive. The origin of the structural changes are expected to be due to chemisorption of PCN to surface atoms on the gold nanoparticles, which is considered using DFT methods (*vide infra*).

### Dependence of SERS Spectrum on Concentration of PCN

**Figure 5:**
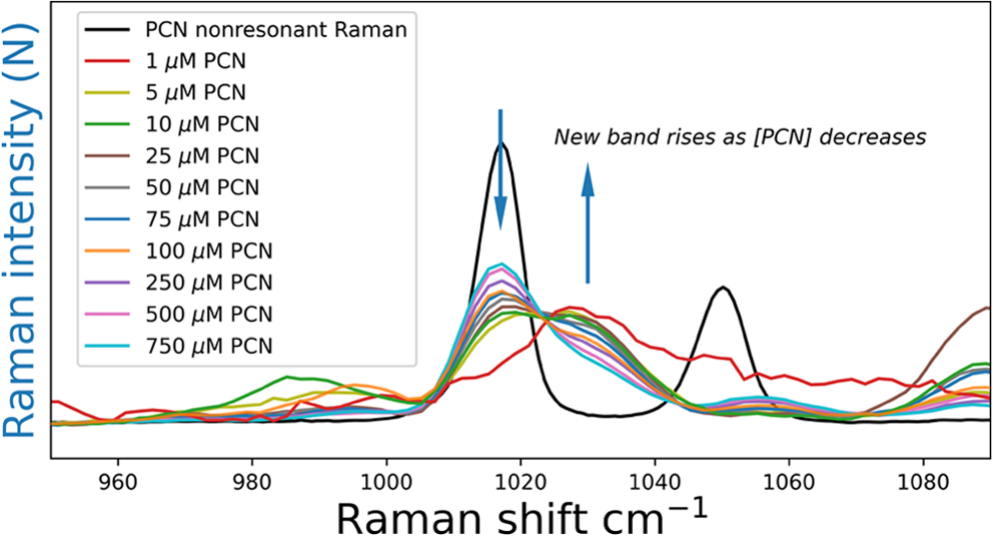
Normalized SERS spectra of PCN, in the range of the ring-breathing band at 1020–1025 cm^–1^. Arrows indicate bands increasing and decreasing with a decrease in PCN concentration. The band at 1030 cm^–1^ increases in relative intensity as the concentration of PCN decreases and is not present in the (black) nonresonant Raman spectrum of neat PCN; it is assigned to a PCN–Au_n_ species, component 3 in Figure 6.

The concentration dependence of the SERS spectrum reveals three distinct species, assigned tentatively as (i) surface bound, (ii) adsorbed to the surface, and (iii) free in solution, near the surface. There are multiple changes and shifts observed in the Raman spectrum when compared to the nonresonant spectrum of PCN. We highlight here the most prominent band of PCN at 1020 cm^–1^, which corresponds to a pyrazine ring vibration. It appears shifted in the SERS spectrum, as a function of the concentration of PCN. When normalized to the area of the band at 1020 cm^–1^, Figure 5, the spectra show an isosbestic point at 1025 cm^–1^, indicating the presence of two species with a Raman band in this range.^12^ Normalization of the band area reduces effects of concentration on the observed intensities and highlights the shift in band position.

At lower concentrations, the ratio of PCN to gold nanoparticles is lower and the relative contribution of the shifted bands is higher. Therefore, the shift is correlated with a greater area of gold surface available per PCN molecule, indicating that the spectrum observed at low concentrations of PCN is due to a PCN-gold(surface) complex.

### Multivariate Curve Resolution (MCR) of **PCN**-SERS Spectra

MCR decomposes the set of spectra into sets of component `spectra’ that can be combined with various weightings to reconstruct each of the spectra in the set. The components are not in themselves actual spectra of individual species present contributing to the acquired spectra. However, they can match closely real spectra when one species dominants the spectrum over a concentration range. MCR was applied to the set of spectra obtained over a range of concentrations of PCN to determine the minimum number of components that the measured SERS spectra can be explained by (i.e., that give a minimum of residuals). Spectra were normalized to the area of the bands between 1000 to 1020 cm^–1^. The MCR analysis indicates one component at high concentrations, and that two components are present in the spectra at low concentrations of PCN, Figure S8.

**Figure 6:**
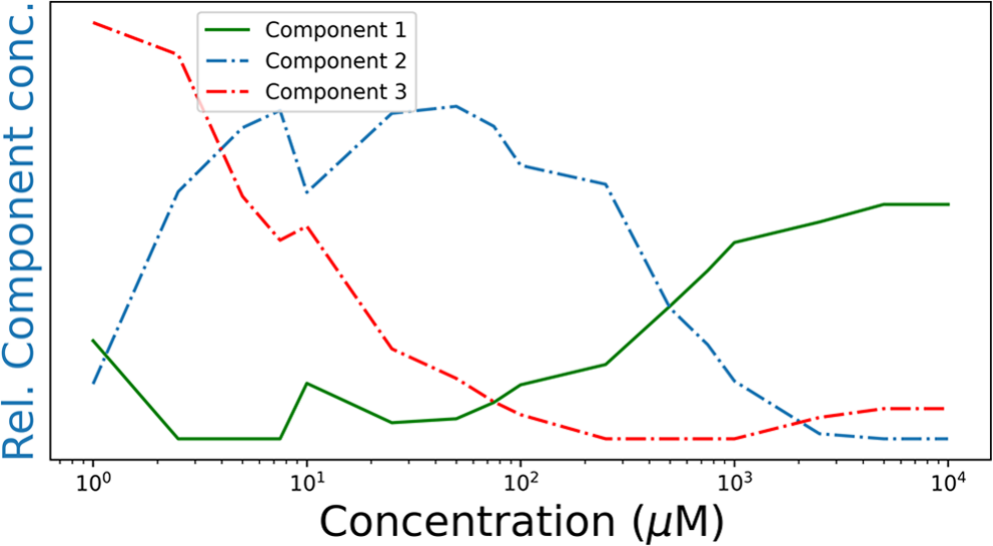
Relative contributions of components in the spectra shown in Figure 5, as a function of PCN concentration. At higher concentrations of PCN, nonresonant Raman scattering is expected to be the main contributor, whereas at low concentrations the relative contribution of a PCN-gold complex is expected to be the highest. The middle component, 2, follows the concentration profile in Figure 3.

The first component corresponds well to the nonresonant Raman spectrum of PCN in water. Raman bands of PCN are not observed at these concentrations in the absence of colloid and hence the bands likely correspond to SERS of nonspecifically adsorbed PCN. The MCR component spectra compare well to the spectra obtained by scaled subtraction of the SERS spectra at different concentrations (Figure S8). The contribution of each MCR component spectrum to the experimental spectra is PCN concentration dependent, Figure 6. The contribution to the Raman spectrum from PCN (nonspecifically adsorbed) decreases with decreasing concentration as expected. The second component, present at lower concentrations of PCN, is likely due to specifically adsorbed PCN. The second component's contribution varies in a similar manner to the overall SERS intensity, Figure 3.

The third component behaves in an unexpected way. Its relative contribution to the total spectrum increases with decreasing concentration of PCN. The only other material present in solution during these measurements are the gold nanoparticles. Therefore, component 3 is likely a species formed by coordination of PCN to gold ions, *vide infra*.

### Dependence of SERS Spectrum on SERS Substrate

Roughened gold surfaces can also yield SERS, and are better suited to compare changes to molecular structure over time, as their surface roughness is static in contrast to the dynamic nature of colloid aggregation.^13,16,25^ The SERS spectrum obtained using an electrochemically roughened gold bead, using the method of Liu and co-workers,^37^ was recorded to compare with SERS spectra obtained with aggregated gold colloids (Figure 4). Similar differences in spectra with the gold bead substrate are observed compared to the nonresonant spectrum of PCN as observed with aggregated gold nanoparticles. The main differences are observed at 650 cm^–1^, 1020 and 1450 cm^–1^, indicated with a dashed blue line in Figure 4. The similarities in SERS spectra suggest that the same molecular structures are present at both SERS substrates.

### PCN-Gold Interaction: Molecular Structure of Bound Species

Raman spectra at various concentrations thus far suggest a dependence of the structure of the observed PCN species on its concentration. At lower concentrations, there is relatively more gold surface available per molecule. At low concentrations, PCN can bind to the gold surface, and it is this species which dominates the SERS spectrum. Coordination of PCN to gold will change its symmetry and bond strengths. These change the intensity and position of bands in the Raman spectrum. Coordination of similar pyrazine- or pyridine-based compounds to gold is known,^38−40^ as are the effects of coordination on Raman shifts of their bands in SERS spectra.^41,42^

However, the bonds between gold and nitrogen ligands are generally labile.^40^ For example, when AuCl_4_^–^ is added to PCN during time-resolved Raman analysis, Raman bands associated with the complex are only observed transiently, Figure S9, but quickly disappear again. Additionally, attempts to prepare a PCN-gold complex with one Au atom bound similarly to the species we expect to see yielded highly unstable crystals. Both of these products yielded Raman scattering characteristic of a complex, but only transiently, decomposing rapidly^43^ under the laser used, Figure S10. In contrast, the measured SERS shifts are highly stable. Therefore, we expect that the PCN species observed at low concentrations is bound to bulk gold more strongly than a single gold atom or ion.

### DFT of PCN and Gold Nanoparticles

The structural change to PCN that is expected to be the cause of the measured changes in Raman spectra is a coordination of PCN to surface atoms on the gold particles. The localization on the surface makes that the Raman scattering of the complex formed is more likely to be enhanced by the surface plasmon, due to the proximity requirements of SERS.^28^ However, interactions with gold can also change the energies of vibrational modes in the molecule and even the normal modes themselves (change in symmetry). Therefore, the Raman spectrum will change also. PCN can bind in any of several manners to gold clusters, e.g., through the nitrile group, and/or either of the pyrazine nitrogens, however, for simplicity only one binding mode is explored in the present study. Binding to the N4 of the pyrazine to gold shows an especially pronounced change in the Raman spectrum in the band near 1450 cm^–1^, corresponding to the in-plane bending mode of the three hydrogen atoms (Figure S11). This vibration in particular is interesting, because it seems to appear in SERS measurements with an increased intensity. A more complete comparison of all calculated modes with the experimentally measured bands can be found in the SI, Table S1.

**Figure 7:**
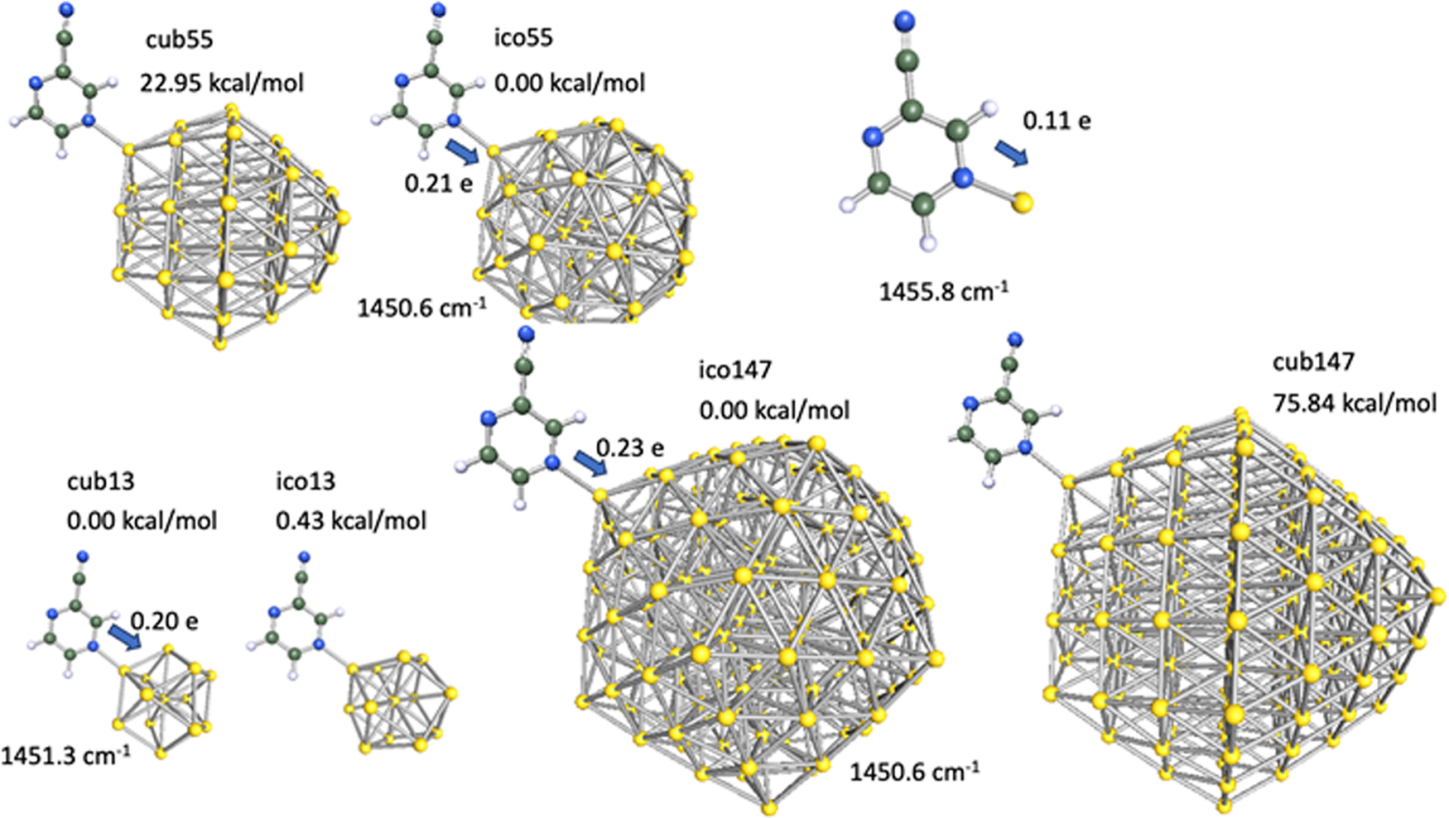
Optimized geometries, relative energies, and vibrational modes for PCN adsorbed on gold nanoparticles with 1, 13, 55, and 147 atoms. Particles with cuboctahedra and icosahedral symmetry are denoted cub and ico, respectively. Vibrational modes and electron density transfer are given only for the ground-state geometries.

We are interested in the dependence of the shift of the vibrational mode at 1455 cm^–1^ on the increase in the size of the gold nanoparticles, and so calculated the wavenumber at the ground state of each moiety. The data are summarized in Figure 7.

Although experimentally unrealistic, the binding of a single gold atom at 4-nitrogen of PCN results in a characteristic vibration at 1455.8 cm^–1^ and charge transfer of 0.11 electron to the gold atom, leaving the PCN slightly electron-deficient. For larger gold clusters, both cuboctahedra and icosahedron gold structures were considered. PCN molecules adsorbed onto a cuboctahedra gold nanoparticle with 13, 55, or 147 atoms showed a –0.43, 22.95, and 75.48 kcal/mol difference in energy compared to PCN molecules adsorbed onto corresponding icosahedron gold nanoparticles, respectively. The characteristic vibration at 1455.8 cm^–1^ is shifted to 1451.3 cm^–1^ for the 13-gold-atom cluster and 1450.6 cm^–1^ for the 55 and 147 gold-atom-clusters. Similarly for the 13 gold-atom-cluster, a charge transfer from PCN to the cluster of 0.20 electrons was observed, with charge transfer of 0.21 and 0.23 in the case of the 55 and 147 gold-atom-clusters, respectively. The shift in band position converges to 1450.6 cm^–1^ for a 55-atom nanoparticle with diameter of 1.1 nm. The frequency shift correlates well with the electron transfer between the molecule and the particle. Detailed Mulliken analysis, Figure S12, shows gradual depletion of electron density at the three hydrogen atoms with the increase in particle size. We assume that the reduced electron density corresponds to reduced force constants at the C–H bonds, leading to the shift to lower wavenumber in the Raman spectrum. Ultimately, larger-sized gold adducts yield larger shifts in peak position and more shared electron density. From this, we infer that the large shift and stabilized signals are originating from an adduct comprising of PCN and gold nanoparticles.

### Albumin Quantification Using SERS of PCN

**Figure 8:**
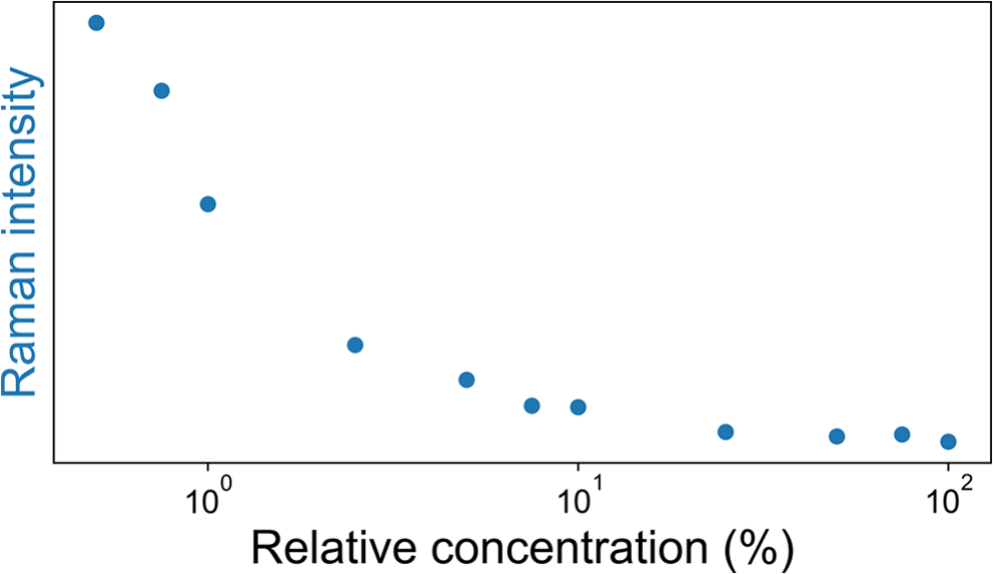
Albumin calibration curve: SERS intensities at various relative concentrations of albumin, starting at 44 μM. A constant amount of nanoparticle solution and PCN were used in each measurement. Intensity was calculated from the integrated surface area of a vibration of PCN.

The SERS spectrum of PCN shows contributions of both PCN itself and gold-bound PCN species. While SERS intensity changes nonlinearly with concentration, the intensity of the bands correlate well with concentration. This correlation allows for the quantification of PCN at concentrations below 100 μM. For the current assay, PCN was kept at a concentration of 50 μM. SERS intensity was recorded after adding varying amounts of albumin, up to the physiologically relevant concentration^33,34,44^ of 44 μM, Figure 8, as also tested with fluorescently (FITC) labeled albumin, Figure S14. The amount of added albumin is too low to affect the aggregation of the nanoparticles, Figure S6, and hence the decrease in intensity is consistent with the available concentration of PCN decreasing upon addition of albumin. Spectra used in preparing the calibration curve are shown in Figure S13.

### Quantifying Albumin from Cell Studies

Control experiments using fluorophore-labeled albumin show that the albumin permeating the cell barrier reaches 8% of the initial (apical) concentration. This permeability results in an approximate concentration of 3.5 μM in the basolateral solution (Figure S16).

**Figure 9:**
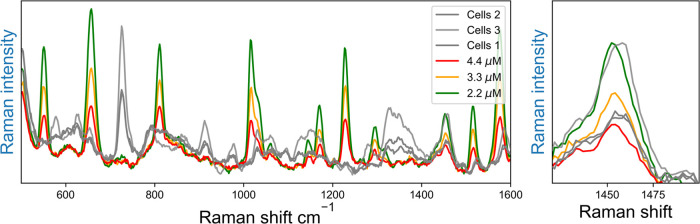
SERS spectra of PCN at 50 μM using nanoparticles as SERS substrate, (colored) from the calibration curve, compared to (gray) spectra obtained from cell media (λ_exc_ 785 nm). Expansion of the Raman bands at 1450 cm^–1^ (right) show intensities with the cells to be between the relative intensities of 10 and 5%. See Figure S15 for full spectral range.

The basolateral solution from permeation experiments performed with nonlabeled albumin were collected for SERS analysis. PCN was added to these solutions and allowed to equilibrate and the Raman (SERS) spectrum was then recorded, Figure 9. The amount of albumin present was determined, using the band at 1450 cm^–1^, to be ca. 3.5 (between ca. 2.2–4.4) μM, which corresponds well with permeation of albumin determined using fluorophore-labeled albumin. Standard addition of 3.5 μM albumin was carried out to further verify the quantification, and resulted in the expected suppression of signal also, Figure S17, without affecting other bands in the spectrum.

The SERS spectra obtained from cell media show a reduced intensity for most bands associated with PCN, as well as the appearance of new bands. These new bands likely originate from compounds in the cell medium, and compounds excreted by the cells, such as adenine (724 cm^–1^), which has a low limit of detection in SERS, showing strong Raman scattering.^45^ Unfortunately, some of these new bands overlap with the bands of PCN. Bands associated with PCN that is not bound to the gold surface are both obscured due to bands of other compounds, and also reduced in intensity due to competition with the other molecules for the surface of the gold, and hence enhancement.

Notably, the band at 1450 cm^–1^, which is associated with PCN bound to gold, remains relatively unchanged by the influx of contaminants from the cells. We think the relatively strong bond of the PCN-gold surface complex helps the molecule to stay on the surface, when in competition with biomolecules from the cells.

## Conclusions

Quantitative SERS spectroscopy of PCN was demonstrated at physiologically relevant concentrations. The differences observed both between the nonresonant Raman and SERS spectrum of PCN, and as a function of PCN concentration, indicate that the SERS spectra report different ratios of three distinct species over different concentrations of PCN. The assignment of spectral changes to changes in chemical structure was assisted by MCR analysis and DFT calculations for unbound PCN, adsorbed, and surface-bound PCN.

Addition of albumin to the PCN-containing solution results in competition for binding PCN between albumin and the gold surface. With a constant [PCN], the decrease in PCN SERS intensity reflects the amount of albumin present in the solution. This method was applied to determining the amount of albumin that permeated the HUVEC layer to be collected on the basolateral side, providing the same concentrations as observed with conventional, FITC-labeled albumin analyzed using fluorescence spectroscopy.^35^

The effect of biomolecules on aggregation poses a limit to the applicability of the assay to biological matrices without sample pretreatment. Approaching the upper limit for quantification (50 μM in the present case) should be avoided, Figure 3. For analysis of concentrated matrices, dilution and some pretreatment may be necessary before use of nanoparticle aggregation-based SERS analysis. However, in relatively simple/clean buffered solutions, such as is often the case with organ-on-a-chip systems, the method described here could suffice.

Besides the contents of the medium, the data indicate selection criteria for the choice of analyte. At lower concentrations, primarily surface-bound PCN is observed, while spectra from calibration data show contributions from nonbound PCN at higher concentrations. However, in the spectra obtained from cell-exposed medium, almost no contributions from nonbound PCN were observed despite the similar concentrations involved. This observation of only bound PCN in complex media measurements indicates that binding is an important factor in observing the analyte. DFT calculations indicate that the pyrazine/pyridine ring nitrogen plays the largest role in this binding, and hence for many drug-like compounds the present approach is likely to be of relevance.

The use of an alternative SERS substrate, a roughened gold-bead surface rather than the gold nanoparticles, shows potential to work over a broader concentration range, since aggregation is not required, albeit that the signal intensities are lower. Both the SERS substrate as well as the concentration of sample can be tuned, so that the analyte to be measured falls in the most sensitive concentration range for the measurement. We anticipate that this label-free method will find use in more sophisticated analysis of tissue barriers and in vitro models. Examples include in-flow evaluation of endothelial cell layers on a chip, under conditions involving disease-inducing factors that change endothelial permeability, or portable albuminuria sensors based on SERS.

## Supporting Information Available

Experimental methods, discussion of calibration curve and Transwell insert experiments and additional data and spectra (PDF)
