# Supplementary material for: Exploring Surface-Enhanced Raman Spectroscopy of Pyrazine-2-Carbonitrile for Indirect Label-Free Albumin Quantification in an In Vitro Endothelium Permeability Assay
Source: Anal Chem. 2025 Feb 10;97(7):4075–83. doi: 10.1021/acs.analchem.4c05906 (PMC11866285; doi:10.1021/acs.analchem.4c05906)
Supplement: Supplementary file 1 — ac4c05906_si_001.pdf [file ac4c05906_si_001.pdf]

Supporting information

Exploring Surface-Enhanced Raman Spectroscopy of Pyrazine-2-Carbonitrile for Indirect Label-free Albumin Quantification in an *in vitro* Endothelium Permeability Assay

W. J. Niels Klement,[a,b] Daniel R. Duijnstee,[b] V. Telle,[b,c] Aleksandar Staykov,[d] Wesley R. Browne,[*,b] and Elisabeth Verpoorte[*,b]

[a]Molecular Inorganic Chemistry,Stratingh Institute for Chemistry, Faculty of Science and Engineering, University of Groningen, Nijenborgh 3, 9474AG Groningen, the Netherlands

[b]Pharmaceutical Analysis, Groningen Research Institute of Pharmacy, University of Groningen, Antonius Deusinglaan 1, 9713AV Groningen, The Netherlands

[c]Present address: Department of Psychiatry and Neurochemistry, Institute of Neuroscience and Physiology, University of Gothenburg, Blå stråket 15, vån 3, SU / Sahlgrenska, 413 45 Göteborg, Sweden

[d]International Institute for Carbon Neutral Energy Research (WPI-I_2CNER), Kyushu University, Japan

email: w.r.browne@rug.nl, e.m.j.verpoorte@rug.nl

	Page
Experimental	S3
Albumin solutions and calibration curves for reference experiments	S3
Gold nanoparticle synthesis	S3-S4
Cell studies	S5
Transwell inserts for HUVEC experiment	S6-S7
Interference by cell components	S8
Albumin quantification positive control	S9-s10
Reference Raman spectroscopy of PCN	S11
Multivariate Curve Resolution (MCR)	S12
Transient observation of PCN-Au with time-resolved Raman spectroscopy	S13-S15
Density Functional Theory	S16-S18
Albumin permeability in HUVEC cells	S19
Raman spectra during albumin permeability determination with standard additions	S19-S21

Experimental

Albumin solutions and calibration curves for reference experiments

Calibration curves were prepared for fluorescence analysis, using fluorescein labeled albumin. This was obtained as a pre-made solution, 20 mg/mL, from Sigma. This solution was divided into aliquots (200 μL) and frozen. Before each experiment an aliquot was thawed. The highest concentration used in cell studies, 0.2 mg/mL (44 μM) was taken as a maximum point for the calibration curve. From here, further dilutions were made by adding amounts of the stock aliquot to PBS, to 0.25% (0.11 μM) of the initial 44 μM concentration, with 10 samples for each calibration curve. The samples were exposed to as little light as possible during the preparation, to avoid photo-bleaching. After mixing, the samples were transferred to a quartz cuvette with a 0.1 cm pathlength and measured in an Edinburgh Instruments Fluorimeter. An excitation scan was performed to determine the ideal excitation wavelength of 455 nm. Using 1 nm slits for both detection and excitation, 1 nm step size and 500 ms dwell time, a spectrum was obtained between 470 nm and 600 nm to reveal the emission band of the fluorescein label. Measurements were performed three times and accumulated, and the data was processed afterwards using Spectragryph12. The resulting integrated signal intensities were plotted versus concentration. A trendline was added with the equation $y = 117138x + 60541$ and $R^2 = 0.9879$ using Microsoft Excel.

Preparation of SERS substrates

While citrate-reduced nanoparticles also yielded reproducible spectra, ascorbic acid-reduced nanoparticles were found to yield the highest SERS intensities with the current method. Therefore, ascorbic acid-reduced gold nanoparticles were used for all measurements. All glassware used in preparation of gold nanoparticles, for degassing solvents and for SERS measurements, including cuvettes, was cleaned with aqua regia before use.

64 mg of $HAuCl_4$ was dissolved in 500 mL of degassed water in a three-necked-round-bottom flask equipped with a reflux condenser, and brought to a boil. The apparatus was then wrapped in aluminum foil and the light switched off for the duration of the procedure, as colloids can be sensitive to light (Langer, J. et al. ACS Nano, 2020, 14, 28–117.) While stirring vigorously, 7.5 mL of 10 weight % L-ascorbic acid was added to the boiling mixture. During the addition, a color change from colorless to orange to deep red was observed. The mixture was boiled for another 30 min, after which the heat was removed and the solution was allowed to cool down to room temperature in the dark. When cool, the entire mixture was degassed again in vacuo using a membrane filter draining straight into a brown glass bottle wrapped with aluminum foil for storage. The colloidal mixture was then used without further purification. The obtained nanoparticle solutions were characterized using transmission electron microscopy (TEM), Figure S1 and UV/vis absorption spectroscopy. The nanoparticles used are relatively spherical in shape and are polydisperse ranging in size from ca. 10 nm to ca. 50 nm. When stored in the dark, the gold nanoparticles were stable for several months without significant aggregation, degradation, or change in SERS intensities obtained. Before use, the storage bottle was gently swirled to homogenize the nanoparticle suspension, after which an appropriate amount was poured out in a clean glass vial. A same batch of gold nanoparticles was used for experiment sets to facilitate comparison.

Electrochemically roughened gold surfaces were prepared by repeated electrochemical oxidation and reduction cycles to roughen the surface.(Liu, et al. Mater. Chem. Phys. 2002, 73, 129–134.) Their preparation and properties were discussed in detail earlier.(Klement, et al. Langmuir 2023, 39, 10383–10394.)

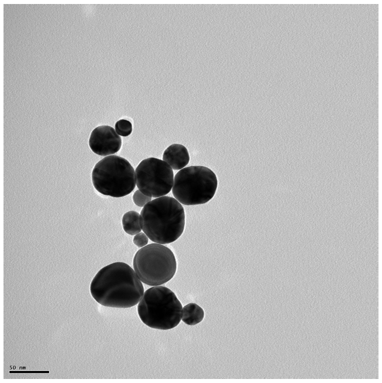

Figure S1: TEM micrograph of gold colloids used in SERS experiments. The scale bar is 50 nm.

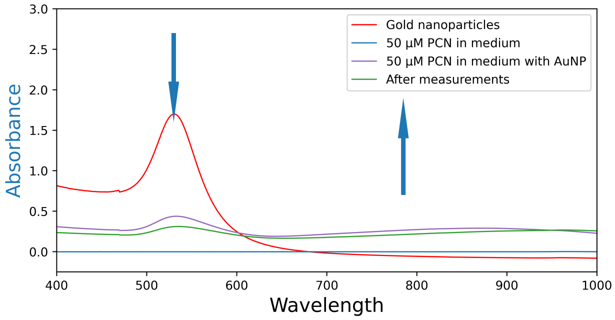

Figure S2: UV/Vis absorption spectra of various solutions containing PCN, medium and colloids. Upon aggregation of the colloids, a color change from red to a blue-ish purple is observed. This is manifested in the absorption spectrum as a decrease of absorbance in the blue and increase in the red. The absorbance increases over the range 700 to 1000 nm where, in the present study, excitation at 785 nm and the accompanying Raman scattering occurs.

Cell studies

Human umbilical vein endothelial cells (HUVEC) were used in cell studies. Opting for a tissue-barrier model facilitates comparison of the SERS method presented here with a fluorescence-based method of albumin detection. Cells were obtained from the University Medical Center Groningen, the Netherlands (UMCG). Cells between passage 5 and 7 were used for these experiments. Cells were cultured with EGM-2 medium (Lonza Biologics, Basel, Switzerland) until an appropriate cell count was reached. Albumin permeability studies were carried out using Transwell inserts (Gibco, ThermoFisher Scientific, USA) with diameter of 1 cm, pore size of 1-2 micron, and pore density of 2×10^6 cm^{-2}, Figure

S3 and Figure S4. Gelatin (0.1% in water) was added to the apical side of the membrane surface. HUVEC cultures were seeded onto the insert on top of the gelatin gel layer, at approximately 100,000 cells cm^{-2}. Medium was refreshed after 24 h. Confluence was reached after 2-3 days, confirmed optically by observing the cell layer under a microscope. Confluence during the measurement in the Transwell experiments was confirmed by adding a slightly greater volume of liquid in the insert compared to the well plate. In non-confluent inserts, the volume of the liquids level out due to the imbalance in pressure. Confluence of the cell layer was confirmed by maintenance of the difference between the liquid levels, Figure S5.

During albumin transport studies, albumin (44 μM in Hank's balanced salt solution, HBSS) was added on the apical (top) side of the cells, which is approximately the concentration of albumin in the umbilical vein.(Hoppe, et al. Transfusion Medicine and Hemotherapy 2009, 36, 353–354 and Anupriya, et al. Ind. J. Child Health 2024, 10, 77–79.) This solution was incubated on top of the cells for 30 min, after which the basolateral (bottom) side liquid was collected for analysis, Figure 2.

Transwell inserts for HUVEC experiment

During the permeability experiment, 1 mL of liquid was added to both the apical and basolateral side. Due to the smaller radius of the Transwell insert, 1 mL of liquid results in a higher liquid level on the apical side. The confluent cell layer maintains this difference in liquid level. Control plates without cells show rapid equilibration of liquid levels.

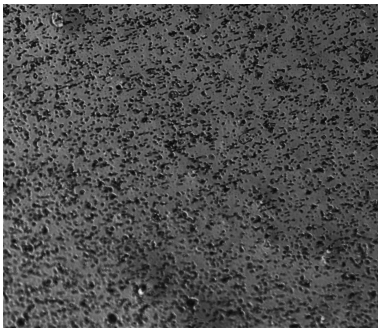

Figure S3: Photo of the base of a Transwell insert showing pores of 1-2 micron.

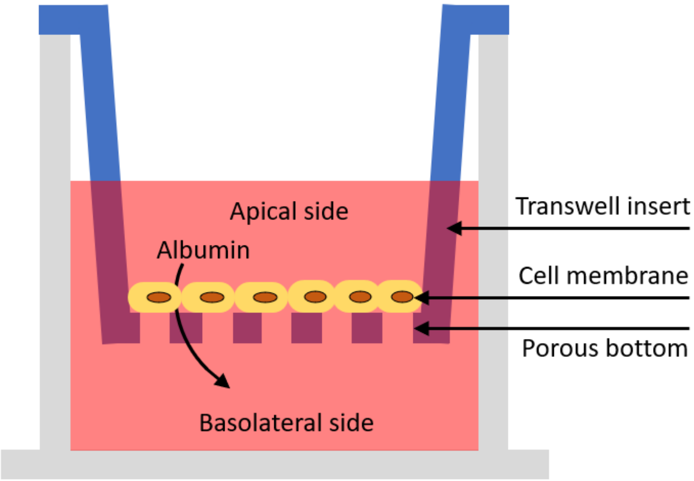

Figure S4: Transwell inserts made of PET sterile plastic were suspended in a well plate. The porous base facilitates cell growth when coated with hydrogel (0.1% gelatin in water in this case). Cell medium is added on both the apical and basolateral-side for incubation. When the cell layer is confluent, the apical-side medium is spiked with albumin (to 44 μM). Cell medium on the basolateral side is collected for analysis after 30 min.

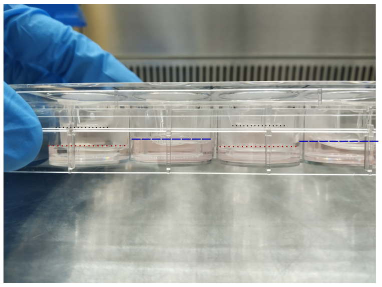

Figure S5: 12-Well plate containing Transwell inserts used in experiments with HUVEC cells. From left to right, rows 1 and 3 contained a confluent cell layer and on rows 2 and 4 were not seeded with cells. The gelatin hydrogel coating layer was present on all Transwells. The approximate height of the medium meniscus in the well and inserts is indicated with a dotted line. Black is the cell medium level in the wells with cells. Red is the medium level on the basolateral-side, in the wells with the cells. Blue is the equilibrated cell medium level in the wells without a cell layer.

Interference by cell components

A challenge poised by the high sensitivity of SERS to small molecule species is that signals originating from sources other than the analyte of interest may be enhanced also, as discussed by Fornasaro and colleagues (Fornasaro, S. et al. Anal. Chem. 2020, 92, 4053–4064.) in a recent inter-laboratory study. Furthermore, earlier studies have shown that a standardized procedure does not exclude contamination in SERS measurements.(Klement, et al. Langmuir 2023, 39, 10383–10394.) Issues with reproducibility make comparisons between measurements, and by extent quantification studies, one of the major challenges in the SERS field.(Langer, J. et al. ACS Nano, 2020, 14, 28–117.) Hence, control experiments to elucidate and fully understand the SERS spectrum are required when desired analytical concentrations are low.

The effect and role of other biomolecules present during SERS analysis is also to be considered. At high concentrations, biomolecules, and in particular proteins, can interact with gold nanoparticles to form a corona around them.(Monopoli, et al. J. Am. Chem. Soc. 2011, 133, 2525–2534.) This corona can potentially hinder aggregation, and by extension SERS enhancement factors. The effect is observed in Figure 3, where high concentrations of PCN hinders aggregation, resulting in lower signal intensities. However, the corona can be formed by albumin or other proteins as well,(Monopoli, et al. J. Am. Chem. Soc. 2011, 133, 2525–2534.) which are often present in biological matrices or cell medium. Therefore, HBSS (Hank's Balanced Salt Solution) is used in the Transwell plate studies here. It is not expected that a concentration of albumin, or other compounds that interact strongly with gold, higher than 50 μM, is obtained from the cells.(Milo, R. BioEssays 2013, 35, 1050–1055; Cooper, G. The Molecular Composition of Cells; Sunderland (MA): Sinauer Associates, 2000; p NBK9879; Sender, et al. PLoS Biology 2016, 14, 1–14.) Estimations exclude such contributions in the current system; the HUVEC layer is 3-5 micron thick and accounts for less than 0.015 percent of the total volume in the experiment, even in the unlikely event that all cells become apoptotic. The cell layers consists

of 70 % water,(Cooper, G. The Molecular Composition of Cells; Sunderland (MA): Sinauer Associates, 2000; p NBK9879; Feijó Delgado, et al. PLoS ONE 2013, 8, e67590.) and the cell contents can result in approximately 40 μM of solute, assuming the molecular weight of a small molecule, 1000 g/mol. However, proteins are most likely to interact with gold, and given an average molecular weight of 50 kDa,(Hendil, et al. J. Mol. Biol. 2002, 315, 627–636) yield a total molarity closer to 1 μM, which corresponds relatively well to other estimations,(Milo, R. BioEssays 2013, 35, 1050–1055) and is insufficient to disrupt colloid aggregation. We confirmed this further, with a positive control containing 44 μM albumin, Figure S6. Therefore, disruption of aggregation is not expected during the current experiments. Aggregation was also assessed for SERS measurements by UV/vis absorption spectroscopy, Figure S2.

It could be that permeation of the endothelial layer by 44 μM albumin would be too high to quantify accurately, when measured together with 50 μM PCN and possible contributions from the *in vitro* tissue using the current configuration, which is aimed at 1-15 μM albumin. For higher concentrations, a dilution of the analyte solution might be appropriate.

Albumin quantification positive control

As seen with UV/vis absorption spectral analysis, colloidal aggregation is not hindered by the addition of albumin. Indeed, albumin should then not hinder overall SERS intensities. This was verified by measuring the spectrum of ruthenium(II) tris-bipyridine ($[Ru(bpy)_3]^{2+}$) with and without albumin present. The addition of albumin has little effect on the observed band intensities.

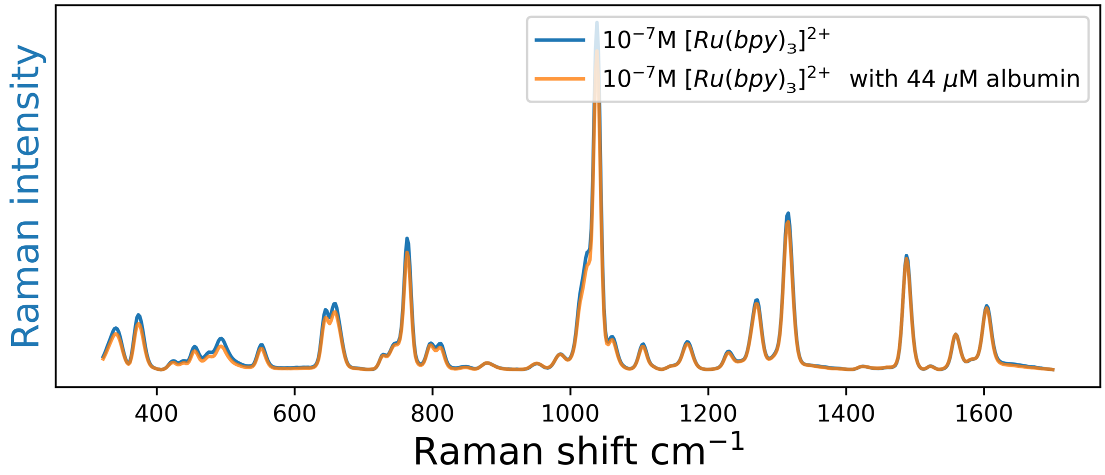

Figure S6: SERS spectra of strong SERS scatter $[Ru(bpy)_3]^{2+}$ (0.1 μM) with and without 44 μM albumin, with the same amount of gold colloid.

Reference Raman spectroscopy of PCN

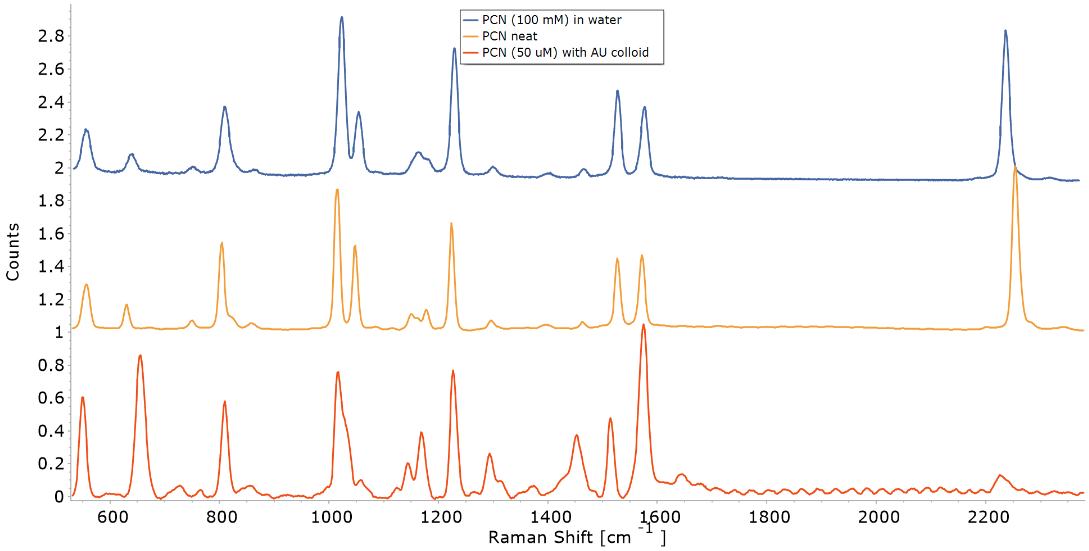

Figure S7: Raman spectra of PCN (neat), PCN in water at 100 mM and SERS spectrum of PCN (50 μM. Minor shifts of band positions but significant broadening are observed between neat and aqueous PCN. Large differences between the non-resonant Raman spectra and the SERS spectrum are observed.

Multivariate Curve Resolution (MCR)

The component spectra yielded by the MCR analysis were plotted against experimental data of corresponding concentrations of PCN, Figure S8. The first component resembles the non-resonant Raman spectrum of PCN. The second component resembles the SERS spectrum of PCN. The third component has bands at the shifted locations observed in Figure 4. It is expected that this component resembles the spectrum of a PCN-Au species.

Even at the lowest concentration measured in this dataset (5 μM) there is still a mixture of both PCN-SERS species, as indicated also by the relatively broad band at 1020 cm^{-1} compared to the sharp bands of the single species. Effectively. the measured spectrum is a mixture of component 2 and 3. The MCR component assigned to PCN-Au, component 3, indeed shows a sharper band at 1030 cm^{-1}, a shift in the same direction as expected from the data in Figure 5.

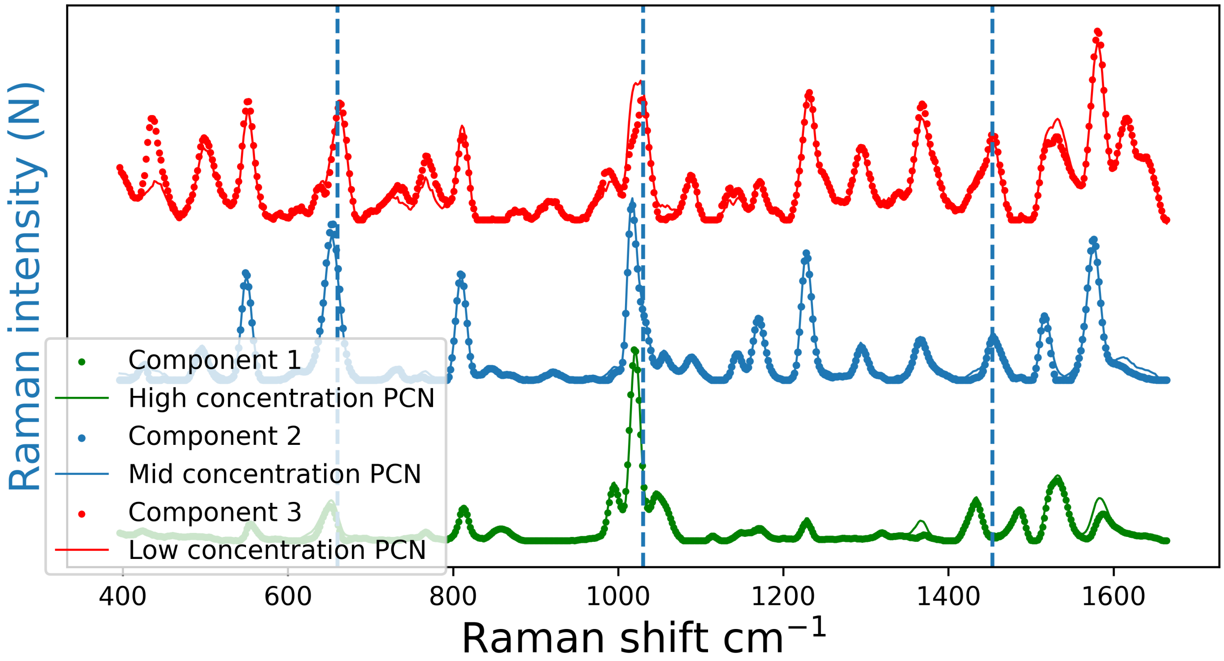

Figure S8: Comparison of components suggested by MCR software and recorded spectra of PCN at corresponding concentration. Component 3 (dotted line) is expected to be an estimation of the spectrum of PCN-gold. Characteristic bands are indicated with a vertical dashed line. The lowest concentration SERS spectrum contains contributions from both component 2 and 3, visible by, e.g., the broad band at 1020 cm^{-1}, whereas the MCR components have sharper bands at this location, that are slightly shifted from another.

Transient observation of PCN−Au with time-resolved Raman spectroscopy

Gold is the expected to be the cause of the observed shifts between Raman and SERS spectra. In the SERS spectra, unshifted bands are also observed in most cases, suggesting a mixture or equilibrium of species present during SERS measurements. It is expected that this mixture is PCN SERS and SERS of a PCN-gold complex. Since the gold-nitrogen bond can be relatively labile, we use time-resolved Raman spectroscopy at a roughened gold bead with fast (sub-second) acquisitions and low (>1 mW) laser power to identify transient formation of species observed in the SERS studies with gold colloid.

Time-resolved SERS measurements were recorded continuously from a solution containing 1 mM PCN with SERS enhancement was provided by a roughened gold bead as expected.

While stirring gently, 0.25 equivalent [250 μM] of $KAuCl_4$ was added to the PCN solution (at t = 2 s). After addition, a small shift in the band at 1020 cm^{-1} was observed transiently, for ca. 1.5 s, with rapidly decreasing intensity, Figure S9. Furthermore, new bands were observed at 1450 cm^{-1} and 1500 cm^{-1} were observed for the duration of a single exposure (500 ms). Prominent shifts are indicated with blue dashed lines. It is noted that for colloidal SERS measurements the PCN-gold species is expected to be more localized on the surface, rather than coordinated to a gold ion, however the resulting shifts obtained with the addition of $KAuCl_4$ could still give a meaningful insight on the influence of gold on the vibrational structure of PCN; similar shifts are produced. Furthermore, when a gold complex such that with PCN is close enough to a bulk gold surface, such as the SERS active electrode, the gold ion in the complex can interact with the bulk gold, and form an add-atom to the surface. We expect that this process contributes to the transient nature of our observations, as the surface interaction could potentially destroy the PCN-gold(III) interaction.

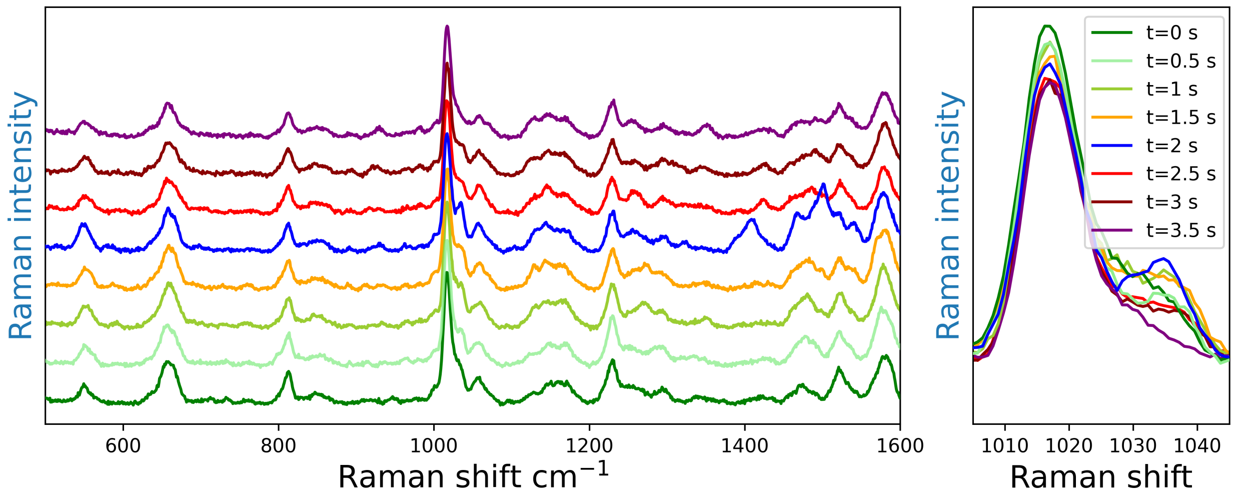

Figure S9: SERS spectra of PCN at 1 mM in water, using a roughened gold bead for surface enhancement and excitation at 785 nm. Exposure time for each spectrum in the time series was 0.5 s. $KAuCl_4$ (0.25 mM) was added at t = 3 (blue spectrum), after which brief transient changes are observed in the Raman spectrum. For example, the band at 1400 cm^{-1} and the shoulder at 1030 cm^{-1}. Prominent shifts are indicated with blue dashed lines. (right) expansion of spectrum at ca. 1020 cm^{-1} shows the shoulder more clearly.

The white crystalline solid product obtained upon mixing of $KAuCl_4$ with PCN was characterised by Raman spectroscopy. The product was light sensitive and degraded upon contact with the laser, resulting in melting of the crystal and apperance of black dots in the formed liquid. Measuring with short exposures ($>$500 ms acquisition time), and low laser power $>$1 mW provided a spectrum, Figure S10, showing mostly contributions from PCN, and a several new bands that correspond to bands observed in the SERS spectra, Figures 4 and S9.

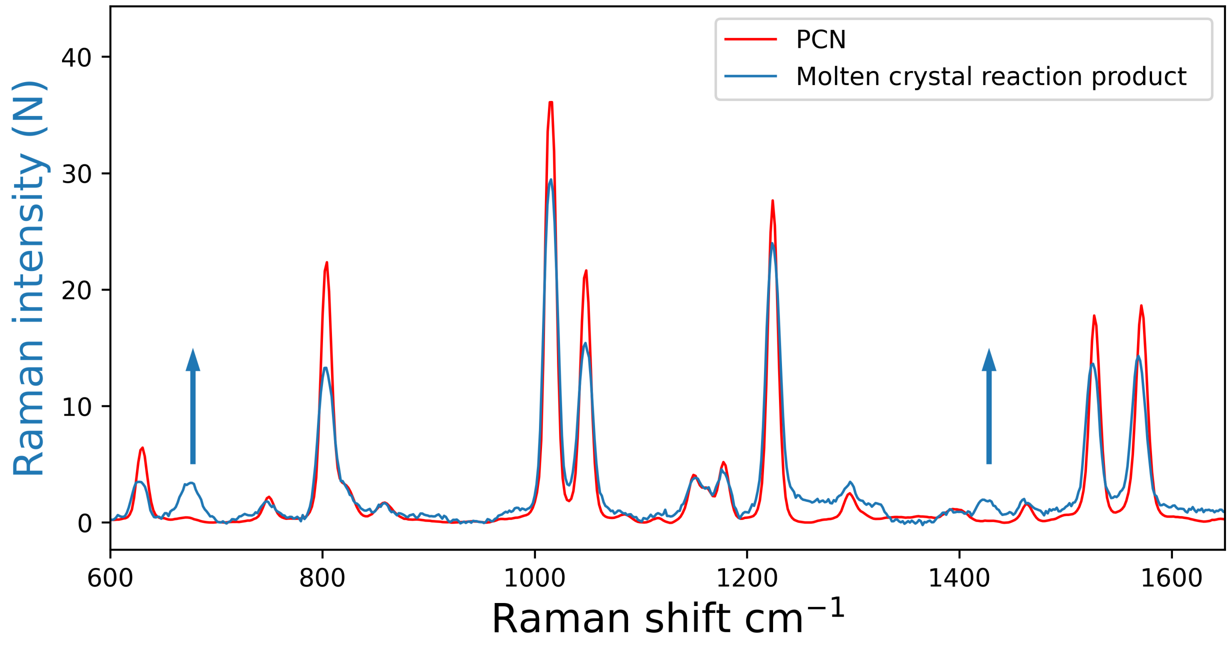

Figure S10: Raman spectra (785 nm) of PCN and the solid obtained from the reaction between PCN and $KAuCl_4$. Arrows indicate new bands present after the reaction.

Density Functional Theory

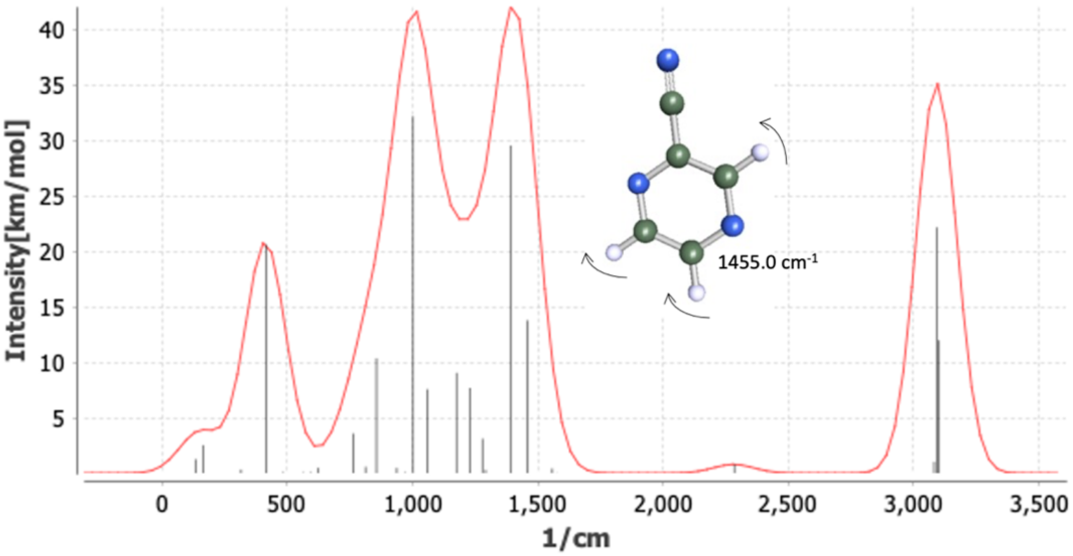

Figure S11: Computed IR spectrum of pyrazine-2-carbonitrile with the displacements of the mode at 1455 cm^{-1} shown.

Table S1: Comparison of band positions in wavenumbers between those calculated by DFT and measured for PCN and PCN bound to gold. Bands near the same Raman shift corresponded to a similar vibrational mode for each vibration for PCN and PCN bound to gold (PCN$-$Au$_n$). Shifts in the calculated spectra correspond to shifts in the measured spectra, in the same direction, for each shifted band.

PCNAU	E(harm)	E	PCN	Calc. Intensity	SERS	SERS	Raman spectrum		
h stretch	3196.761	3155.75	x		low conc/comp	high conc			
h stretch	3191.9	3148.53	x						
h stretch	3161.157	3140.67	x						
cn stretch	2275.685	2266.45	x						
ring vib in plane	1575.144				1620	1620			
		1570.62	x	big	1575	1580	1575		mixture
ring oscillation in plane	1513.416	1529.75	x	medium	1520	1520	1525		
para h wag + ring breath	1465.768	1469.18	x	medium	1452	1450	1465		
h wag sym ortho in plane	1414.562	1406.76	x	weak/only IR	1375	1375	1400		
h wag sym para in plane	1304.137	1303.81	x	medium			1300		
in plane ring breath/deform	1243.278	1232.19	x	medium	1290	1280	1275		shoulder
in plane ring deform	1214.851	1214.36	x	weak/only IR	1230	1225	1225		
in plane n1 wag	1163.472	1178.63	x	medium	1169	1175	1180		shoulder
					1140	1140	1155		shoulder
ring breath in plane+n1	1072.605		x	medium	1080	1080			shoulder
		1057.07				1055	1050		
in plane sym ring deform pa	1015.084	1010.7	x	medium	1027	1016	1016		
h-flap asym	988.999	993.87	x	weak	986	980	too weak		
h-flap sym	931.719	954.4	x	weak	924	925	too weak		
h-flap sym	859.211	865.2	x	weak	846	870	860		
ring breath/deform in plane	819.3	803.27	x	medium		820	810		
					767	767			
ring wag/flap out plane	738.379	757.86	x	weak	738	725	750		
ring breath in plane+CN	663.101			medium		660			shoulder
		627.56	x				630		
ring deform out plane	583.605	572.46	x	weak		shoulder	560		
ring deform in plane+CN	562.022	561.27	x	medium		550	550		
ring deform in plane	478.526	480.62	x	med/low		500	480		shoulder
n-ring torsion/flap out plan	427.164	405.77	x	weak/only IR		440	420		

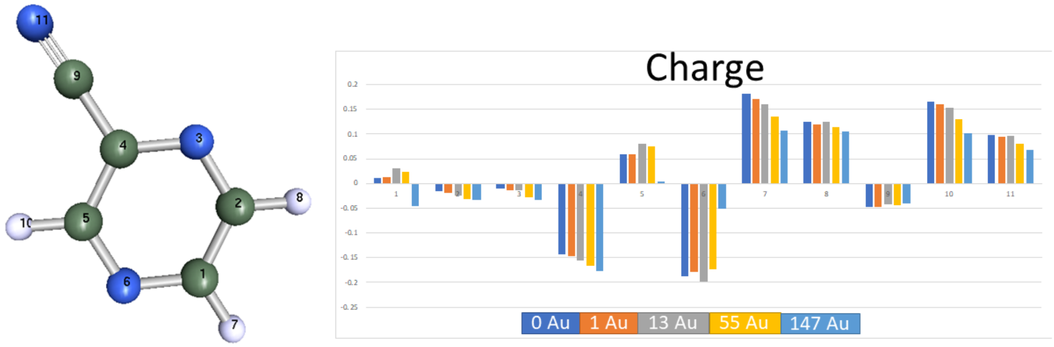

Figure S12: Mulliken analysis of electron density from DFT studies. Positive and negative values denote more or less electron density, respectively, on the atoms associated with the number in the figure on the left. The largest differences are observed for atoms that participate in the highlighted vibrational mode at 1450 cm^{-1}. It is expected that this band is shifted partly because of these changes.

Albumin permeability in HUVEC cells

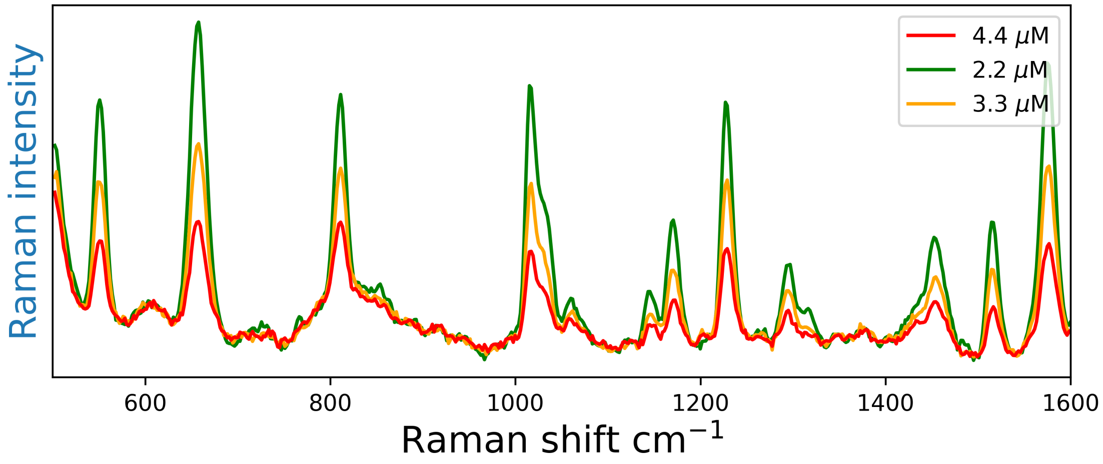

Figure S13: SERS spectra of PCN (50 μM) with various concentrations of albumin, with constant amount of gold colloid.

Raman spectra during Albumin permeability determination with standard additions

The method of standard additions was used to verify the amount of BSA determined, and the expected decrease was observed adding a known amount of albumin to the unknown amount in the basolateral cell medium. The intensity of the band at 1450 cm^{-1} is reduced and roughly corresponds to that expected for ca. 7 μM, Figure S17.

The recorded intensity for a cell sample without standard addition corresponds to between 3.3 and 4.4 μM, whereas for the sample with an additional 3.5 μM added provides an intensity corresponding to between 4.4 and 11 μM.

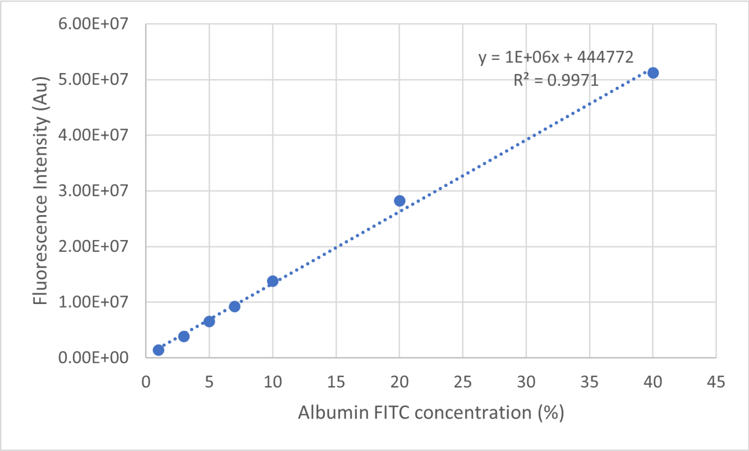

Figure S14: Calibration curve for Albumin-FITC, from area of the fluorescence spectrum of the fluorescein label. The abscissa is percent concentration with respect to 44 μM (albumin).

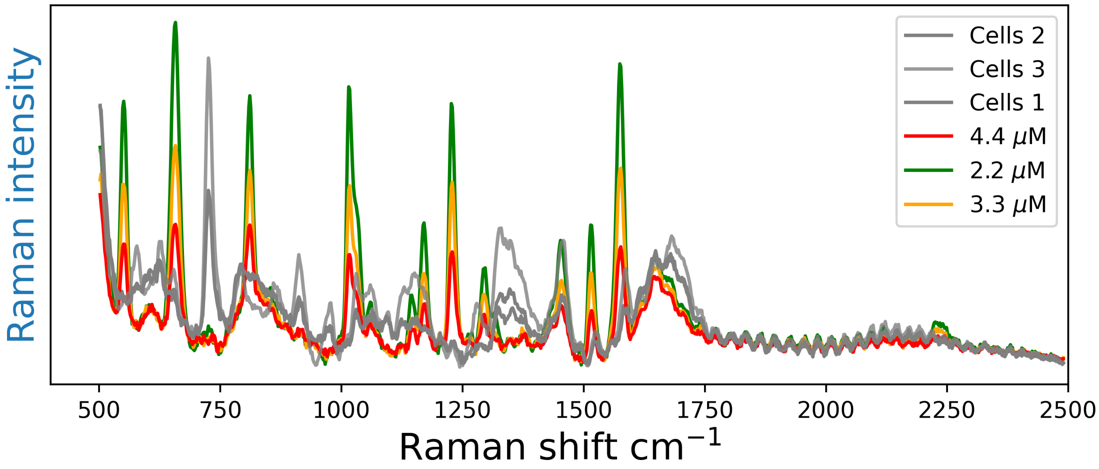

Figure S15: Full range of spectra shown in Figure 9

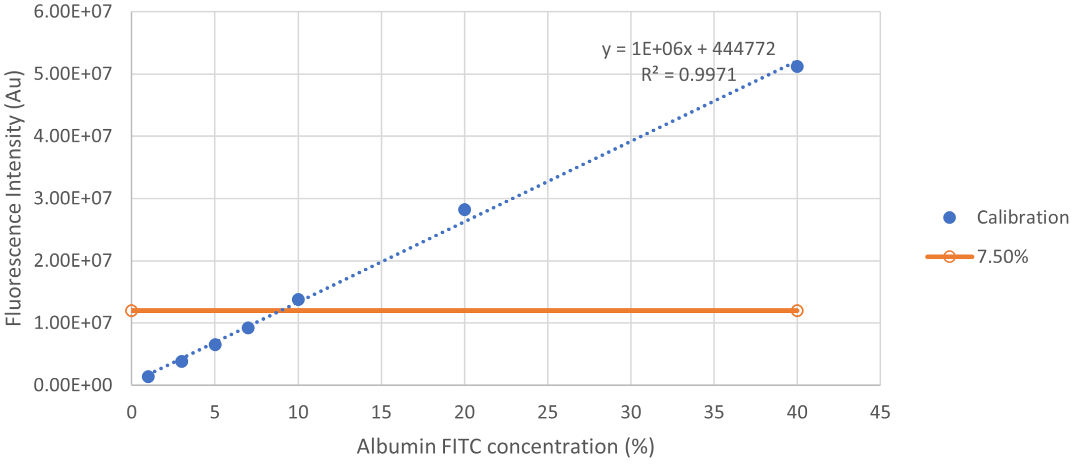

Figure S16: Calibration curve for fluorescently labeled albumin prepared on the same day as the HUVEC permeability experiment. The abscissa is percent concentration with respect to 44 μM (albumin). The intersection of the calibration curve with the averaged intensities from the fluorescence spectra recorded on the basolateral side of the transwell inserts after the HUVEC permeability experiment is at 7.5%, and corresponds to 3.5 μM albumin on the basolateral side (crossed over).

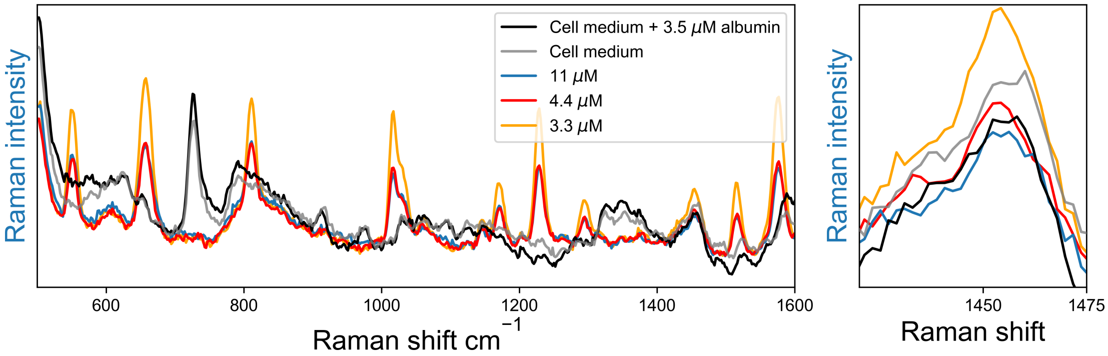

Figure S17: SERS spectra of PCN (50 μM) with various concentrations of albumin, with constant amount of colloid. Colored spectra are from calibration measurements. Gray spectrum is from cell studies, and black spectrum is a cell sample with addition of 3.5 μM albumin. The intensity of the band 1450 cm^{-1} is decreased in accordance with expectation from the calibration curve, and lies in between 4.4 and 11 μM, expected for the new amount of albumin of 7μM. Other bands are not affected by the standard addition.
